# Harnessing the Immunogenic Potential of Gold Nanoparticle-Based Platforms as a Therapeutic Strategy in Breast Cancer Immunotherapy: A Mini Review

**DOI:** 10.3389/fimmu.2022.865554

**Published:** 2022-03-31

**Authors:** Xiao-Yang Chen, Lin-Yue Lanry Yung, Puay Hoon Tan, Boon Huat Bay

**Affiliations:** ^1^Department of Anatomy, Yong Loo Lin School of Medicine, National University of Singapore, Singapore, Singapore; ^2^Division of Pathology, Singapore General Hospital, Singapore, Singapore; ^3^Department of Biomolecular and Chemical Engineering, College of Design and Engineering, National University of Singapore, Singapore, Singapore; ^4^Duke-NUS Medical School, Singapore, Singapore

**Keywords:** adaptive immunity, breast cancer, gold nanoparticle, immune response, immunogenic cell death, immunotherapy, innate immunity, tumor microenvironment

## Abstract

Breast cancer remains the most common malignancy among women worldwide. Although the implementation of mammography has dramatically increased the early detection rate, conventional treatments like chemotherapy, radiation therapy, and surgery, have significantly improved the prognosis for breast cancer patients. However, about a third of treated breast cancer patients are known to suffer from disease recurrences and progression to metastasis. Immunotherapy has recently gained traction due to its ability to establish long-term immune surveillance, and response for the prevention of disease recurrence and extension of patient survival. Current research findings have revealed that gold nanoparticles can enhance the safety and efficacy of cancer immunotherapy, through their unique intrinsic properties of good biocompatibility, durability, convenient surface modification, as well as enhanced permeability and retention effect. Gold nanoparticles are also able to induce innate immune responses through the process of immunogenic cell death, which can lead to the establishment of lasting adaptive immunity. As such gold nanoparticles are considered as good candidates for next generation immunotherapeutic strategies. This mini review gives an overview of gold nanoparticles and their potential applications in breast cancer immunotherapeutic strategies.

## Introduction

As reported in GLOBOCAN 2018, breast cancer has the second highest incidence globally, with an incidence rate that has been increasing in both developing and developed countries in comparison with other cancers (1). Breast cancer is much more prevalent in women (2), and contributes most of the cancer-associated morbidity and mortality for females worldwide (3).

Breast cancer is characterized by uncontrollable neoplastic growth in the breast (4), through a multistep tumorigenesis process involving many complex molecular mechanisms (5). This heterogenous cancer may arise from environmental influences, genetic alterations, metabolic changes, or a combination of these factors (6, 7). Although there are many morphological variants of breast cancer, the most common is ductal carcinoma which encompasses about 80% of all diagnosed breast malignancies (8). Ductal carcinoma can be further classified by their molecular subtypes based on the expression levels of estrogen receptor, progesterone receptor, and human epidermal growth factor receptor 2 (HER2) (9–13). Different prognoses have been reported for various breast cancer subtypes, and therapeutic strategies that target these subgroups were therefore developed accordingly (14, 15).

The success rate for breast cancer therapy is largely determined by the point of detection, where the overall mortality rate can be reduced by 20% with early diagnosis (16). The inception of mammography has significantly increased early detection of breast malignancies among females, as clinical and self-examinations for the detection of early abnormalities in the breast have always been challenging (17).

Conventional treatments for breast cancer, like chemotherapy, radiation therapy (RT), and surgery have significantly improved both disease-free survival and overall survival (OS) (18, 19). Unfortunately, post-treatment recurrences of this disease remain a formidable challenge for clinicians (20). Immunotherapy has recently emerged as a promising anti-cancer strategy for prolonged survival in breast cancer patients (4). This novel anti-tumor approach allows the activation and stimulation of the host immune system to recognize and eradicate tumor cells, as well as establish a long-term immunological memory to prevent tumor recurrences. However, this relatively new form of therapy has its own fair share of clinical safety concerns and efficacy issues (20). Lately, the concept of incorporating nanotechnology into cancer immunotherapy, has also been gaining traction (21). Because of its multiple surface functionalities and unique properties, gold nanoparticles (AuNPs) are actively being researched as cancer diagnostics and therapeutics (22). This mini review seeks to highlight the perspectives and potential of AuNP-based platforms as a strategic approach in breast cancer immunotherapy.

## Current Treatment for Breast Cancer

Breast cancer diagnostics and therapeutics have made considerable progress in the last decade due to better understanding of disease pathogenesis (23). Current treatments for breast cancer are chemotherapy, RT, and surgery. Although these approaches have been widely employed by clinicians for decades, they have their own limitations and challenges (24). Chemotherapy uses cytotoxic drugs, like 5-fluorouracil, anthracyclines, carboplatin, cyclophosphamide, and taxanes, to eliminate rapidly proliferating cells that include both tumor and normal cells (like immune and epithelial cells) (25). Although the survival rate for breast cancer patients under the age of 50 years, can be improved (by up to 15 years) by 10% and those older by 3% (26), the non-targeted nature of chemotherapy brings unwanted systemic damage to the host body (24). RT employs high doses of radiation, such as γ-rays and X-rays, to destroy tumor cells and shrink tumor size. Like chemotherapy, it also damages healthy cells and tissues near the treatment area (27). Surgery removes tumors that are accessible, large, and resectable (24). Although the prognosis is highly favorable when early diagnosis of breast cancer is met with conventional treatments (14), nearly a third of these patients still suffer from disease recurrences and progression to metastasis (18).

Compared to conventional chemoradiotherapeutic approaches, the emergence of cancer immunotherapy is attributed to its potential to provide better prognosis and prevent disease recurrences (20). Immunotherapy is designed to enhance and/or restore the host immunity to seek and eliminate cancer cells (28). Although more immunotherapeutic drugs and combinatorial treatment strategies involving immunotherapies have been approved lately (29), there are still issues with autoimmunity and systemic inflammation (30), as well as challenges in targeting solid tumors (31). Fortunately, the incorporation of nanotechnology can potentially enhance both the efficacy and delivery of these immunotherapeutic drugs (32).

## Nanoparticles in Breast Cancer Nanomedicine

Nanotechnology is defined as the manipulation of materials with dimensions from 1 nm to 100 nm (33). Due to their small physical size, nanomaterials offer unique physical and chemical characteristics that are distinct from their bulky counterparts (34). NPs can be generally classified under organic NPs or inorganic NPs (35).

Organic NPs, like liposomes, polylactic-co-glycolic acid (PLGA) NPs, and extracellular vesicles (EVs), are known to have high drug delivery efficiency and low toxicity (36). Liposomes were the first nanomedicine approved for clinical use (37), and their application in breast cancer has been gaining popularity (38, 39). Liposome has an outer lipid layer that can be modified to mimic the biophysical properties of the host cells, and a core carrying its cargo, usually chemotherapeutic agents (40, 41). Unlike its non-biodegradable predecessors, PLGA NP is a clinically approved copolymer made of lactic and glycolic acids, which can be hydrolyzed into normal metabolites under physiological conditions, and has excellent *in vivo* biocompatibility (42, 43). Like liposomes, PLGA NPs are used as nanocarriers for breast cancer therapy. EVs are double-layered phospholipid vesicles secreted by cells (44), and can be categorized into apoptotic bodies, exosomes, or microvesicles (45). Doxorubicin (DOX)-loaded into exosomes could be used for breast cancer treatment due to enhanced tumoricidal activity and reduced cardiotoxicity (46). More information on the use of organic NPs in anti-cancer therapies can be found in the review paper by Karpinski and co-workers (44).

Inorganic NPs, such as carbon nanotubes (CNTs), quantum dots (QDs), and metallic NPs, have higher modifiable surface area to volume ratio that can be easily functionalized (47, 48). Discovered in 1980s, CNTs are rolls of graphene that can be categorized as either single-walled or multi-walled (44). In addition to being good nanocarriers due to their unique biological, chemical, and physical properties, they can also be used for photothermal therapy (PTT) when exposed to near-infrared radiation (NIR) (49). QDs are nanoscale semiconductors that exhibit a quantum confinement (50), and have high photostability with a broad spectrum of absorption and narrow emission bands, allowing them to be mainly used as an imaging modality and/or a therapeutic agent for photodynamic therapy (PDT) and/or PTT (51). However, the main limitation for QDs is the lack of an optimized production process (44). For metallic NPs, AuNPs have been widely studied for use in breast cancer diagnostics and therapeutics (21, 52). Hence, their potential to be incorporated into the next generation of breast cancer immunotherapeutic strategies will be discussed in the subsequent sections.

## Anti-Cancer Properties of Gold Nanoparticles

AuNPs can easily be synthesized from the reduction of gold salts (21), into variable sizes and shapes including decahedron, hexagon, icosahedron, octahedron, sub-octahedron, nanocage, nanoprism, nanorod, nanosphere, and nanostar (53, 54). Smaller sized AuNPs allow for greater tissue distribution, deeper tissue penetration, and increased cellular internalization (55). This is largely attributed to the phenomenon, known as the enhanced permeability and retention (EPR) effect, where the nano-sized effect of AuNPs allows for greater accumulation in tumor tissues than healthy tissues, due to their extravasation into the tumor microenvironment (TME) through the wide fenestrations of the angiogenic vascular architecture, as well as the lack of normal lymphatic drainage (56). Hence, this permits AuNPs to achieve passive targeting to tumor tissues *in vivo*.

The toxicity of AuNPs is also dependent on their shape, size, surface charge, surface chemistry, coating, and contaminants (57). The cytotoxic feature of AuNPs is attributed to their ability to induce oxidative stress (24). There are studies that report AuNPs are non-toxic although there are conflicting reports that state otherwise (58). AuNPs are popular for diagnostic and therapeutic uses due to their unique catalytic, electronic, magnetic, optical, physical, and structural properties (21, 22, 52, 59). Chemicals, drugs, natural products, and/or probes can bind to the negatively charged surfaces of AuNPs to potentiate their therapeutic efficacy (60–64).

## Gold Nanoparticle-Based Targeted Therapy

Targeted therapy has been deemed safer than traditional therapies, as the latter usually result in collateral damage to healthy cells and tissues due to their non-specificity. Thus, breast cancer patients are expected to suffer less side effects from targeted therapies (4). Trastuzumab (Tz) or Herceptin is the first humanized monoclonal antibody (mAb) to be approved for routine clinical use as targeted therapy for HER2^+^ metastatic breast cancer in 1998 (65, 66). HER2 is responsible for cell proliferation in normal cells but an overexpression of these receptors can promote unrestricted cell growth and eventually tumorigenesis (67). HER2 can be neutralized, internalized, and downregulated upon the binding of Tz, with concomitant upregulation of p21Waf1 and p27Kip1 (68). Tz is also known to cause antibody-dependent cellular cytotoxicity on its target cells (69), whereby cells marked with Tz can be targeted for cell lysis by natural killer cells (70).

Interestingly, AuNPs can be conjugated to Tz to allow specific active targeting to breast tumor cells. This concept has been successfully demonstrated in both *in vitro* experimentation and using a subcutaneous MCF-7 human breast cancer murine model (71). Although there was an increase in targeting efficiency for AuNPs that were conjugated with two mAbs for *in vitro* experiments, *in vivo* results showed that one mAb per AuNP (5NP-1Tz) produced the best tumor homing and protracted therapeutic efficacy (71). By conventional wisdom, one may think that a higher number of mAbs on a AuNP can confer superior binding ability. However, a high density of mAbs per AuNP may introduce steric hindrance that reduces the access to its target antigen (71). Hence, it is important to adjust the number of mAbs conjugated per AuNP to achieve the desired active targeting efficacy. Additionally, the same *in vivo* study has shown that long-term intra-tumoral retention of 5NP-1Tz produced continuous therapeutic effect over time (71).

## Immunotherapy with Gold Nanoparticles as Delivery Vehicles

Owing to their EPR effect, AuNPs are able to specifically accumulate in tumor sites and lymph nodes, which allow them to be highly effective delivery vehicles for immunological reagents (30). They are also easily synthesized into different sizes and shapes, to modify properties such as cytotoxicity, distribution, immunogenicity, and metabolism, for various therapeutic uses (20). The high molecular density on their surface and ease of modifications allow conjugation of different molecules like polyethylene glycol (PEG) and arginine-glycine-aspartic acid tripeptide, to improve the overall pharmacodynamics and pharmacokinetics of AuNPs and its cargo (72). This phenomenon has already been elegantly and successfully demonstrated in an *in vivo* study, where chimeric peptides (glycopeptide sequence from mucin-1 and T cell epitope P30 sequence) were attached to the PEGylated AuNPs (PEG-AuNPs), to be used as an anti-cancer vaccination strategy against MCF-7 human breast cancer cells (73).

AuNPs can also function as protective delivery vehicles for compounds that have low intracellular accumulation, such as siRNA, and/or vulnerable to intrinsic enzymatic degradation (5). Thus, the incorporation of AuNPs as nanocarriers in the drug delivery strategy, not only helps with tumor site targeting, but can also improve the solubility and stability of their sensitive cargo and prolong the half-life (74). Studies have demonstrated the potent immunomodulatory effects of *Ganoderma lucidum* polysaccharide (GLP) on the maturation of dendritic cells (DCs) (75, 76). However, due to their low prescribed dosages and rapid clearances, the efficacy of GLP for clinical applications remains questionable. One study showed that the half-life of GLP in the blood can be extended and their accumulation in immune organs can be increased, when AuNPs were used as delivery vehicles for GLP (GLP-Au) (77). Compared to free GLP, the *in vitro* results in the same study have revealed a stronger DC activation and T cell response by GLP-Au. Moreover, combining GLP-Au with DOX produced stronger inhibitory effect against 4T1 murine tumor growth and pulmonary metastasis than free GLP with DOX. Additionally, the activated DCs were also observed to induce both CD4^+^ and CD8^+^ T cell expansion in the spleen.

## Radiation Therapy with Gold Nanoparticles

RT is one of the least invasive standard of care that has been prescribed for over half of cancer patients (78–80). RT exerts cytotoxic and cytostatic effects *via* DNA damage by fractionated focal irradiation (81). However, these irradiations are unable to differentiate tumor cells from normal cells which lead to unnecessary destruction of surrounding healthy tissues (82). Additionally, its tumoricidal ability is often limited by the maximum deposited dosage and that has always remained a challenge among radiologists (83). Hence, there is always a clinical interest to specifically maximize the RT dosage on the tumor and minimize the collateral damage to the neighboring healthy tissues (84).

Fortunately, metal radiosensitizers, such as AuNPs, can increase the sensitivity of tumor cells to irradiation due to its high atomic number granting high absorption coefficient (85–87). Generation of reactive oxygen species (ROS) can be induced by the Auger electron production from the surface of AuNPs (20). By coupling with an active targeting strategy or by the passive targeting of the EPR effect alone, using AuNPs as radiosensitizers allows the reduction of total RT dosage whilst increasing the local RT dosage to tumor sites (82). For example, conjugation of Tz to PEG-AuNPs (Tz-PEG-AuNPs) allowed intracellular uptake of AuNPs into SK-BR-3 human breast cancer cells, which then proceeded to cause DNA double-strand breaks by 3.3 to 5.1 times more than RT alone or RT with PEG-AuNPs (88).

**Figure 1:**
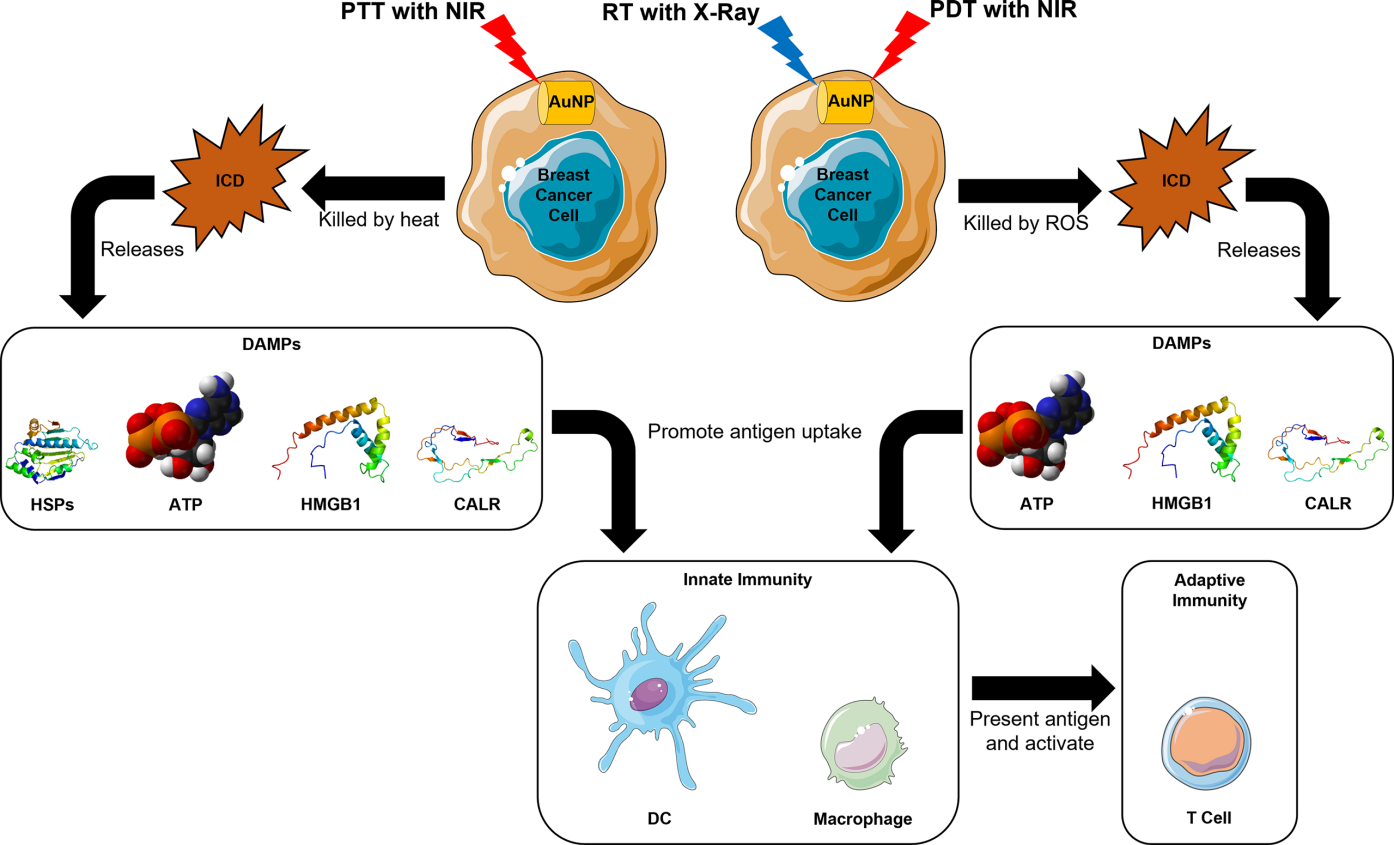
Mechanistic insights for induction of host immune system in AuNP-based RT, PDT, and PTT in breast cancer.

The resultant cell death from RT, known as immunogenic cell death (ICD), can release tumor antigens and improve the host response to cancer immunotherapy through antigen presentation, cytokine secretion, and naïve T cell activation (89–95). Additionally, signals for anti-tumor immunity like adenosine triphosphate (ATP), nuclear protein high-mobility group box-1 (HMGB1) and calreticulin (CALR) are produced upon ICD (92, 96). The release of these danger-associated molecular patterns (DAMPs) help to recruit and activate antigen-presenting cells (APCs) of the innate immune response like DCs and macrophages, and also promote antigen uptake and presentation to CD8^+^ T cells for the establishment of long-term adaptive immunity (96, 97) (**Figure 1**). ATP interacts with purinergic receptor P2X 7 on DCs and activates the NOD-, LRR- and pyrin domain-containing protein 3 inflammasome for proteolysis, maturation and secretion of interleukins 18 and 1β (98). HMGB1 engages toll-like receptor 4 on macrophages to enhance the release of proinflammatory cytokines (99, 100), whereas CALR binds to the CD91 receptors on phagocytes to promote phagocytosis of dying and dead cells (101). This process is especially useful against immunologically cold tumors, like triple negative breast cancer, because the increase in TME immunogenicity was found to be correlated with better prognosis due to improved patient responses to immunotherapy and chemotherapy (102). RT with 14 nm AuNPs showed enhanced RT efficacy, and induced ICD that resulted in significant macrophage infiltration, and hence improved OS in an MDA-MB-231 human breast cancer murine model (82).

## Photodynamic Therapy with Gold Nanoparticles

PDT is a cancer therapy that has spatiotemporal selectivity with minimal invasiveness (103). Its mechanism of action is to generate ROS to destroy tumor cells through a photochemical reaction, using light, photosensitizers like AuNPs, and oxygen (O_2_) from tissues (5, 20, 104). Briefly, accumulated AuNPs at the tumor site can be excited by NIR to generate ROS in the presence of surrounding O_2_, which can be devastating to the surrounding tumor cells (20). Just like RT with AuNPs, PDT with AuNPs is also able to trigger anti-cancer immune response through the ICD process (105) (**Figure 1**).

Unfortunately, PDT is often met with poor efficacy due to the hypoxic TME (106). In addition to promoting tumorigenesis and metastasis (107–109), a hypoxic TME is one of the primary reasons to immunosuppression due to the inhibition of T cell infiltration into the tumor site (110, 111). Hence, tackling tumor hypoxia should be considered before employing a PDT strategy (112). Recently, studies have shown that gold nanocages (AuNCs) have PDT potential due to their hollow porous structures (113, 114). An interesting *in vivo* study demonstrated the feasibility of using AuNC@manganese dioxide (AuNC@MnO_2_) for an O_2_-boosted immunogenic PDT in a 4T1 mouse model, in which MnO_2_ shells degrade under low pH and produce large amount of O_2_ to boost the PDT effect in a hypoxic TME (112).

## Photothermal Therapy with Gold Nanoparticles

Similar to PDT, PTT is another non-invasive cancer therapy that utilizes AuNPs and NIR, but the only difference here is the generation of heat to destroy cancer cells (115). PTT is commonly employed to treat breast cancer by selectively ablating tumor cells (5). Due to their high photothermal conversion efficiency, AuNPs can reach an excited state through the absorption of light (78), at which vibrational energy is emitted as heat and can consequently destroy nearby cancer cells (116).

In addition to the ability to generate ICD in tumor cells like RT with AuNPs and PDT with AuNPs (117), PTT also cause heat-induced cytotoxicity (118). As a result, heat shock proteins (HSPs) are released, which can then form HSP-peptide complexes with tumor antigen peptides to enhance phagocytosis of dead cells and antigen presentation by APCs (119, 120) (**Figure 1**).

## Conclusion and Perspectives

**Figure 2:**
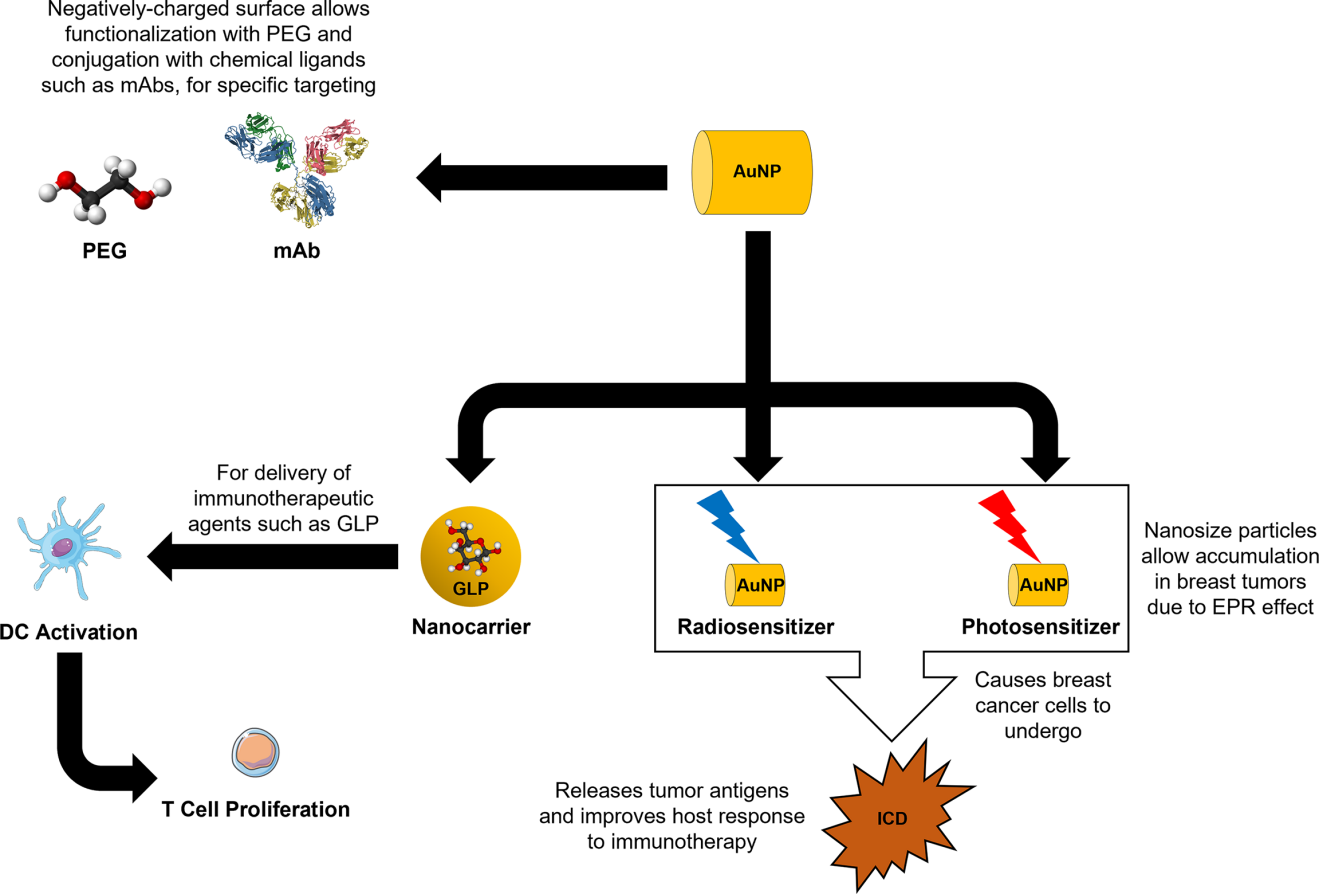
AuNP-based nanooncology approach to breast cancer immunotherapy.

Current research in nanomedicine has shown that AuNPs have excellent biocompatibility and good resistance against degradation under physiological conditions, thus facilitating long-term therapeutic effects in breast cancer patients. Due to their EPR effect and the option to include active targeting strategies, both *in vitro* and *in vivo* studies have demonstrated the promising applications and feasibility of AuNPs in improving the efficacy of various therapies, especially in RT, PDT, and PTT. Additionally, the unique intrinsic properties of AuNPs in stimulating the host immune system through the induction of ICD allows the incorporation of immunotherapeutic strategies. A summary figure depicting a AuNP-based approach as a nanocarrier for immunotherapeutic drugs and stimulation of the host immune system mediated *via* RT, PDT, and PTT is shown in **Figure 2**. Although there is a lot of flexibility in the surface modifications of AuNPs, a re-evaluation of the pharmacology and toxicology of every additional modification will be required, to address any concerns regarding efficacy and safety. More work needs to be done before AuNPs can be routinely used for breast cancer immunotherapy. Nevertheless, recent preclinical data have adequately shown that AuNPs can serve as potential next generation immunomodulators for breast cancer therapy.

## Author Contributions

X-YC wrote the manuscript. BHB, PHT, and L-YLY reviewed and edited the manuscript. All authors approved the manuscript for publication.

## Funding

Kwan Im Thong Hood Cho Temple Professorship to BHB (WBS E-546-00-0042-03).

## Conflict of Interest

The authors declare that the research was conducted in the absence of any commercial or financial relationships that could be construed as a potential conflict of interest.

## Publisher’s Note

All claims expressed in this article are solely those of the authors and do not necessarily represent those of their affiliated organizations, or those of the publisher, the editors and the reviewers. Any product that may be evaluated in this article, or claim that may be made by its manufacturer, is not guaranteed or endorsed by the publisher.
